# Low engagement of key populations in HIV health services in Tanzania: analysis of community, legal and policy factors

**DOI:** 10.11604/pamj.supp.2023.45.1.39591

**Published:** 2023-06-20

**Authors:** Andrew Kigombola, Johnson Lyimo, Mucho Mizinduko, Deogratias Mkembela, Evelyne Maziku, William Kafura, Abubakar Maghimbi, Christine Musanhu, Peter Nsubuga, Zablon Yoti

**Affiliations:** 1 Ciheb Tanzania, Dar es Salaam, Tanzania,; 2 World Health Organization, Dar es Salaam, Tanzania,; 3 Muhimbili University, Dar es Salaam, Tanzania,; 4 United Nations Development Program, Dar es Salaam, Tanzania,; 5 National AIDS Control Program (NACP), Dodoma, Tanzania,; 6 Tanzania Commission for AIDS (TACAIDS), Dodoma, Tanzania,; 7 Global Public Health Solutions, Dar es Salaam, Tanzania

**Keywords:** Key population, access, structural barriers, health services, stigma, discrimination

## Abstract

**Introduction:**

key populations (KP) often face legal and social challenges that increase their vulnerability to HIV. These experiences include criminalization, higher levels of stigma and discrimination which negatively affect access to HIV services. This study aims to understand legal, community and policy factors affecting engagement of KP in HIV health interventions.

**Methods:**

qualitative research key populations design involving a desk review and stakeholder’s engagement. We reviewed program data from NACP on how KP access health services and then conducted three stakeholders’ engagement meetings. Factors affecting access to health services by KP were documented. Data were organized using socio-ecological model (SEM).

**Results:**

program data showed only 49% of the estimated KP accessed health services. Barriers to accessing health services at the interpersonal level included lack of social support and high-risk networks linked with risk behaviours. At the community, stigma and discrimination, limited engagement of influential leaders were noted. In health facilities, lack of trained staff to provide KP friendly services affected utilization of health services. At structural level, despite improvements, still various laws negated engagement of KP such criminalizing drug use, same sex, and sex work. Harassments and arrests further marginalize KP and makes access to health intervention harder.

**Conclusion:**

engagement of key population into HIV health interventions was limited at multiple levels. The study recommends building capacity on KP friendly services for communities, law enforcement and health care providers, further engagement of communities including religious leaders on KP issues and implementing differentiated service delivery models for KP.

## Introduction

The World Health Organisation (WHO) defines key populations (KP) as groups who, due to specific higher-risk behaviors, are at increased risk of Human immunodeficiency virus (HIV) irrespective of the epidemic type or context [1]. Globally the risk of acquiring HIV in key populations is higher than in the general population. The Joint United Nations Programme on HIV/AIDS (UNAIDS) estimated in 2020 that the likelihood of acquiring HIV was 35 times in people who inject drugs (PWIDs), 34 times in transgender women, 26 times in female sex workers (FSW), and 25 times in men who have sex with men (MSM) [2]. Similarly, bio-behavioural surveys in Africa have indicated higher HIV prevalence among key populations; in South Africa, HIV prevalence among MSM ranged from 27% to 47% in 2019 (5). In Malawi, in 2020, higher HIV prevalence was also observed; FSW (50%), MSM (13%), and clients of FSW (12%) [3]. In Tanzania in 2018, HIV prevalence was reported as 2-3 times higher among KP compared to the national HIV prevalence of 4.7% [4]. Across studies, risky behaviors were prevalent, with 29% of PWID shared needles, and condom use was <30% among MSM and < 75% among FSW [4-6].

Key populations behaviors are often criminalized and experience higher levels of stigma and discrimination, which could contribute to underestimation of the disease burden and compromise access to HIV services [1]. Often, they do not have equitable access to HIV prevention and treatment services as the general population. In Ghana, only about 50% of MSM had access to HIV prevention services: stigma and discrimination, negative attitudes by healthcare workers, and lack of KP-friendly services affected the utilization of services [7]. Access to antiretroviral therapy (ART) was low among MSM and FSW when compared with the general population, reported at 13.2% vs 56.55% in Cameroon (12), 31.2% among MSM in Nigeria [8], and 36% among FSW in middle and low-income countries [9]. Assessment of barriers to HIV treatment among FSW and MSM in Uganda found that stigma and discrimination, negative attitudes by health care workers, inadequate skills in managing KP, and criminalization affected access to care and may cause KP to shun services [10]. In Dar es Salaam, only 25% of 578 PWID were regularly seeking care when sick, and only PWID who were employed or knew where to seek care were more likely to be engaged with the health system [11]. Also, screening for infectious diseases such as HIV, sexually transmitted infections (STI), and tuberculosis (TB) was low among PWID [12]. Furthermore, National AIDS Control Program (NACP) data of 2020 showed only 49% of the estimated KP accessed services in Tanzania [13].

Sexual relationships between KP and the general population are common and play a significant role in HIV transmission between different groups [14]. Evidence of overlapping sexual networks between these populations exists; hence, ending the HIV epidemic among key populations is imperative for the success of the national HIV response [15]. This study aims to determine legal, community, and policy factors affecting the engagement of KP in HIV health interventions.

## Methods

**Study setting:** the study was conducted in three zones involving 10 regions in Tanzania in 2022. The zones were Coast, covering Dar es Salaam, Pwani, and Tanga regions. The central zone covers Dodoma, Iringa, Singida, and Tabora regions, and the Lake zone covers Mwanza, Kagera, and Geita regions. National AIDS Control Program data were used to select regions with a high number of KP to participate in the consultations.

**Study design:** the study was a qualitative research design involving a desk review and stakeholders’ engagement meetings.

**Desk review:** we conducted a desk review of global, regional, and national documents such as WHO Consolidated guidelines on HIV prevention, diagnosis, treatment, and care for key populations, the Universal Declaration of Human Rights, and the African Charter on Human Rights. National documents included the health sector, HIV, and the multisectoral strategic frameworks for HIV and the overarching legal frameworks (i.e., the Constitution of Tanzania, the Drug Control and Enforcement Act, and the HIV/AIDS Prevention and Control Act [HAPCA]). The review also included unpublished literature such as minutes and reports of regional stakeholders´ meetings, legal literacy meetings, legal framework assessment, and stigma index conducted by TACAIDS. Key populations program data from NACP were reviewed to understand the regional estimates of KP by type and level of access to services.

**Stakeholders’ engagement meetings:** staff from NACP, TACAIDS, and WHO conducted engagement meetings with stakeholders from the three prespecified zones. The selection was based on familiarity and experience working with key populations. They were oriented on the objectives and expected deliverables of the engagement meetings. The meeting discussion guide, schedule, and presentation slides were developed to ensure standardization. Focus group discussions were conducted in four key participant subgroups: key populations, law enforcement officers, healthcare workers, and religious/community leaders. Participatory approaches were utilized to encourage open and inclusive discussions among KP to understand barriers and drivers to access service at the facility and community level. Team members took extensive notes to ensure all the discussions, themes, and recommendations were captured.

**Participants:** participating stakeholders were purposefully selected to include community and influential leaders, religious leaders, law enforcement and judiciary, community, and health care workers, and KP as beneficiaries.

**Data collection:** a semi-structured guide was developed to capture KP concerns, barriers, and facilitators in improving KP service delivery during the stakeholder engagement meetings.

**Figure 1:**
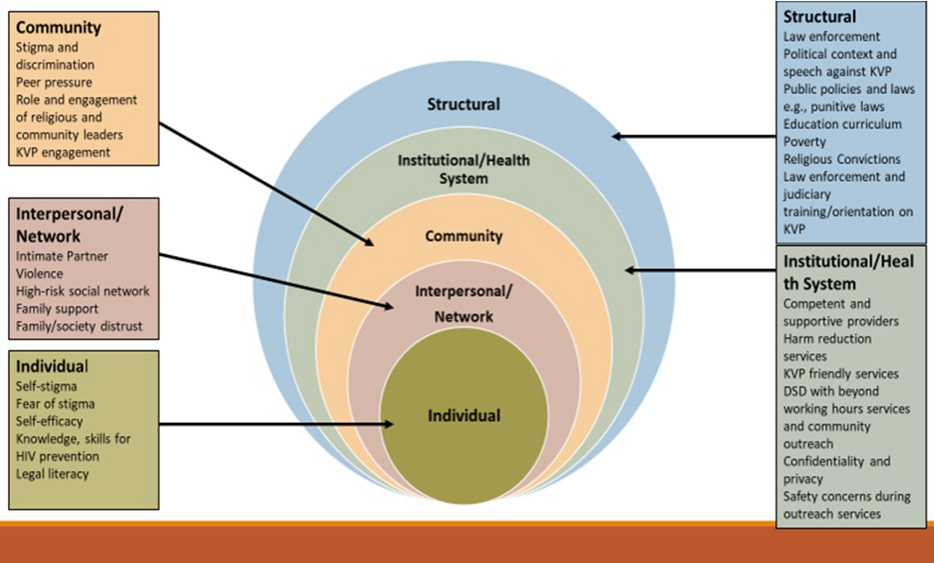
socio-ecological model

**Data analysis:** desk review findings were categorized into the strengths/facilitators and barriers to accessing health services. These were viewed through the lens of human rights and harm reduction. Inputs from the stakeholders’ meetings were coded, and recurring concepts were arranged into thematic areas. The themes were summarised using the social-ecological framework/model (SEM) (Figure 1). This model, developed by sociologists in the 1970s, studies how behaviors form based on characteristics of individuals, communities, nations, and levels in between [16]. The model also addresses complexities and interdependences between socioeconomic, cultural, political, environmental, organizational, and psychological determinants of KP engagement with health services. Thematic analysis was used to group key findings into SEM.

**Ethical clearance and consent:** the stakeholders’ meetings did not collect identifiable data, only views, comments, and inputs were captured. Verbal consent from participants was obtained, and they were informed that no personal data would be collected during the meetings and reports. The assessment was approved by the Ministry of Health and Tanzania Commission on AIDS.

## Results

A total of 64 participants were involved in the stakeholders’ engagement meetings. Table 1 shows a representation of all major groups of stakeholders by type engaged in the meetings. Participants were categorized into the following major groupings; influential community and religious leaders (9), law enforcement and judiciary (13), community and health care workers (8), and KP (34). Various barriers and facilitators to HIV services access were identified during the meetings and desk review. The socio-ecological model was used to categorize these factors.

**Table 1:** participants in the stakeholders' engagement meetings held by TACAIDS and WHO by the zones (N=64)

		Disaggregation					
Zone	Participants	PWID	MSM	FSW	HCW	Police/Judiciary	Influential leaders
Central	15	2	3	4	1	4	1
Lake	22	6	0	7	2	4	3
Eastern	27	2	4	6	5	5	5
Total	64	10	7	17	8	13	9

MSM: males who have sex with men, FSW: female sex workers, PWID: people who inject drugs, HCW: health care workers, TACAIDS: Tanzania Commission for AIDS

Self-stigmatization, fear of external stigma, and low legal literacy among KP were recurring sub-themes under the individual-level theme (Table 2). Self-stigma was high, resulting in low self-esteem and poor self-efficacy in seeking health care services. Key populations (KP) shared their views. “*A friend had rectal gonorrhea, and because it would reveal he was engaging in anal sex; he was afraid to go to the hospital for treatment. He was concerned with being stigmatized and negatively judged by providers and resorted to buying medication in the drug store,” (MSM DSM). “To avoid dry sex with customers, we wish lubricants were available in friendly spots. Buying lubricants in pharmacies is negatively viewed as engaging in anal sex. Some of us use olive or coconut oil for lubrication*,” FSW Dodoma.

**Table 2:** findings at individual (intrapersonal level) and Interpersonal/social networks which negatively affect engagement in health services

Emerging themes (based on SEM)	Sub-thematic groups	Findings
**Individual/intrapersonal level**	Self-stigmatisation	KP perceived/expected stigma and discrimination, resulting in not going to the hospital and resorting to self-medication.
	Fear of external stigma	Double stigma associated with being KP and living with HIV. KP were afraid that their health condition might give away their sexual orientation. Negative judgement by health care workers and law enforcement officers was a barrier to accessing health services.
	Low legal literacy	Most KP were unaware of HAPCA laws and updates in the Drug Control Act. Also, during arrests, the rights of the suspect as per the Criminal Procedure Act were not known by the KP.
**Interpersonal/social networks**	Estrangement by family	Most KP were banished by their families, losing social and financial support and predisposed to HIV risk behaviours (e.g., commercial sex work).
	Distrust of KP	Despite efforts to recover (especially PWIDs), there was constant distrust from family members and communities due to their previous behaviour, such as theft or violence. This lack of full integration into society keeps KP in a vicious cycle of poverty.
	High-risk social networks	Strong social networks were noted, and peer influence was high to propagate high HIV-risk behaviours such as needle sharing and less condom use.

HIPCA: HIV/AIDS protection and control act, KP: key populations, PWIDs: people who inject drugs

External stigma was also high resulting in reduced health service utilization. “*After they (HCWs) knew that I am MSM, they called me into a room full of providers and started questioning why I have an immoral habit. I felt my privacy violated and stigmatized. That was a few years ago. Now there is an improvement in how they provide services, but the feeling of negative judgment still exists*,” MSM Tanga. Under the intrapersonal relationship theme, once discovered as drug users or engaging in same-sex relations, many indicated that they were estranged and distrusted by their families. “*At home, they still lock valuables and bedrooms, thinking I will steal money, jewelry or electronics; they don’t believe I have changed*,” (People who use drugs (PWUD) on methadone, Mwanza). Several themes emerged on how the community impacted the engagement of KP with HIV services (Table 3). All participants agreed that there was high stigma and discrimination towards key populations. With the fear of stigma and discrimination, low health-seeking patterns emerged. *“I wanted to say. These religious leaders, both Christians and Muslims are harsher to MSM and FSW (thinking we chose or decided to become the way we are) and more accommodating to the PWUD,” (FSW from DSM). Religious and community leaders’ engagement was also limited, and they were unaware of any interventions targeting KP. Furthermore, there was no mechanism for reporting discrimination and violence against KP at the community level using street and ward leaders. “We are not engaged in delivering these services or identifying the hotspots which are in our areas. They merely come for a permit for outreach activities but not to involve us,”* (community leader, DSM). Lack of meaningful engagement of KP in the program designing, planning, and implementation was also noted, with KP mostly engaged as peers for outreach activities.

**Table 3:** factors at community levels which hinders engagement of key population in the health services

Emerging themes (based on SEM)	Sub-thematic groups	Findings
**Community**	Stigma and discrimination	High levels of stigma and discrimination toward key populations due to their behaviors. Health care settings, law enforcement and places of worship such as churches and mosques were noted as places with high stigma and discrimination. Key populations felt religious leaders are more tolerant of PWIDS as they view them as victims of the illicit drug trade.
	Religious and community leaders' engagement	Lack of interventions to address stigma and discrimination by community leaders beyond donor-organized sensitization meetings
	KP engagement	Minimal engagement of KP-led or KP-competent CSO at subnational levels (regions and districts) beyond peer outreach services KP were not aware of any economic empowerment programs

CSO: civil and social organization, PWIDs: people who inject drugs, KP: key populations

Under health systems/institutions’ the following gaps were observed; (a) unavailability of competent, user-friendly providers in health facilities, (b) limited differentiated service delivery models for KP, and (c) harassment and safety concerns during the service provision in the hotspots. Interviewed health providers and KP outreach peers reported experiencing arrests and harassment from law enforcement during outreach activities, and drop-in centers were banned as they were thought to promote homosexuality and prostitution. In several cases, community leaders such as street or ward leaders who are unaware of these outreach activities call the police. Other emerging themes are outlined in Table 4. *“I was once caught during a hotspot police raid, and they thought I was a female sex worker despite telling them I was providing HIV testing services. Our supervisor had to come and bail us,”* (peer educator, Geita). “*These drop centers were very useful, offering services for KP in friendly and stigma-free surroundings, but the government decided to ban them,”* (MSM, Tanga). Despite various policies such as National AIDS Policy, Health Sector HIV/AIDS Strategic Plan, and National Multisectoral Framework recognizing KP programming as an important opportunity for ending the HIV epidemic KP still faces myriad barriers and challenges in accessing HIV interventions. Structural barriers include punitive laws that disproportionately affect key populations, economic underdevelopment and poverty, stigma and discrimination, violence, and lack of community empowerment (Table 5).

**Table 4:** institutional/health system factors negatively affecting engagement of key population in health services

Emerging themes (based on SEM)	Sub-thematic groups	Findings
**Institutional/health system**	Unavailability of competent, user-friendly providers in health facilities	KP reported encountering judgmental attitudes and stigma from health care workers during the clinical visits. Only 50% of health care workers are trained to provide KP-friendly services. The presence of KP peers in the facilities eases navigation through the system.
	Unavailability of KP-friendly services beyond normal working hours	KP, especially FSW, preferred evening hours as they work at night and rest during the day. Some larger facilities may have PEP kits at OPD after working hours, but staff are not oriented to provide comprehensive KP services. When faced with arrests, legal aid such as legal representation or bail processing was unavailable after working hours.
	Limited knowledge of the 2008 HIV Prevention and Control Act (HAPCA)	Both community and facility health care workers were either unaware or with insufficient knowledge of the HAPCA. It was the first time most providers learned about the law.
	Harassment and safety concerns during service provision in the hotspots	KP have experienced arrest and harassment from law enforcement during outreach activities under the pretext of loitering or promoting homosexuality and prostitution. Peers' navigators and GBV desks for reporting stigma, discrimination and GBV/IPV were available in facilities but not disaggregated by typology as recommended.

PEP: pre-exposure prophylaxis, GBV: gender-based violence, IPV: intimate partner violence, SEM: social-ecological framework/model, KP: key populations, FSW: female sex workers

**Table 5:** structural barriers affecting engagement of key population in health services

Emerging themes (based on SEM)	Sub-thematic groups	Findings
**Structural**	Punitive laws and policies	The Penal Code criminalizes same-sex, possession of drugs even in small amounts for personal use, and sex work.
	Harassment and arrests from law enforcement	Unwarranted arrests due to the way KP dress or being found in popular spots for sex work and charged for loitering unless they offer bribes/sex.
	Lack of awareness of the protective laws and provisions for KP	Members of the police force, judiciary and health care workers were unaware of some provisions in existing laws i.e., Drug Act (possession of drugs for personal use carries less sentence than trafficking, section 31 grants a magistrate to direct the addict to treatment instead of a jail sentence) and HAPCA protects PLHIV against confidentiality breaches, stigma, and discrimination.
	Few police officers are trained on KP-friendly services	In Dodoma, only 67% of the gender disk officers received training. In Mwanza, only six staff are trained. The greatest training gap was among senior police officers.
	Negative and judgemental religious views	Most religious leaders held negative, judgmental views against KP as immoral and sinners. MSM and FSW were arrested by police force to protect moral decency in the society.
	Differentiated Service Delivery (DSD) Model for KP unsupported by policies	National KP guidelines do not offer tailored services at the community level, such as community ART refills and designated safe spaces for combination prevention interventions for KP.
	Inadequate community empowerment	Limited fund flow to grassroots CSO at regional and district levels. Lack of CSO technical and organisational capacity to apply and manage funds and projects.
	Inadequate stakeholders' engagement for KP	Lack of regular engagement meetings involving law enforcement, KP, religious and community leaders at the regional, implementing partners and council levels.

KP: key populations, CS0: civil and social organization, HAPCA: HIV/AIDS prevention and control act, PLHIV: people living with HIV, MSM: men who have sex with men, FSM: female sex workers, SEM: socio-ecological model

Punitive laws and policies which negatively impact KP health-seeking behavior are summarised in Table 6 and harassment and arrests from law enforcement were commonly reported. “Police routinely harass us in our business areas/spots. If we do not pay them or offer sex, they take us to the police station and open charges,” (FSW, DSM). *“I went to report a case of sexual abuse, and the attending police officer said forced sex is part of the job description as I sell sex. Sometimes it is futile to report,”* FSW DSM. Few police officers were trained in KP-friendly services and victims shared the experience of being denied their ARVs or Methadone while in jail. *“Some police officers have been trained, but still, a huge gap exists. We need more training dealing with gender and sexual violence, also how to address these groups. It is complex as existing laws prescribe punishment,”* (police officer, Dodoma).

**Table 6:** laws adversely affecting and marginalize key population in Tanzania

Some of the laws affecting the key populations adversely		
FSW	MSM	Drug users and PWID
Living partially or wholly on the earning of prostitution or solicitation is a misdemeanour (Penal Code Section 145) Prostitutes loitering or soliciting are fined 500- or a three-month sentence (Section 176)	Having a carnal knowledge against nature or permitting someone to have carnal knowledge a felony with seven years sentence (Section 154) Gross indecency with another male a felony with a five-year sentence (Section 157)	Possession of drugs in small amounts for personal use or possession of drug paraphernalia carries a fine of 0.5-1M or a prison sentence of three to five years. (DCEA section 17) Repeated offenders 10M fine or life in prison

MSM: male who have sex with men, FSW: female sex workers, PWID: people who inject drugs, HCW: Health care workers.

## Discussion

Using the socio-ecological model, our study identified legal, community, and policy-related factors hindering the engagement of KP in HIV health services, with national data showing only 49% of KP were reached with KP-friendly services in 2020. This is consistent with several studies conducted in similar settings. Herce *et al*. found <50% of KP had access to HIV prevention services in Malawi and Angola; furthermore, only 71% were aware of their HIV status [17]. Among transgender in Zambia, low HIV test uptake (22.8%) was noted [18]. In Ghana, about 50% of MSM had access to HIV prevention services: stigma and discrimination, negative attitudes by healthcare workers, and lack of KP-friendly services affected the utilization of services [7]. In Cameroon, access to ART was low among MSM and FSW when compared with the general population (13.2% vs 56.55) (12), 31.2% among MSM in Nigeria [8], and 36% among FSW in middle and low-income countries [9]. Similar findings were observed in studies done in Tanzania; among people who inject drugs (PWID) in Dar es Salaam, only 25% were regularly seeking care when sick [11]. Among MSM living in Tanga, only 32% accessed HIV prevention services such as STI screening and treatment of anal ulcers [19].

At the community level, our study found that stigma and discrimination, minimal engagement of community elders, religious leaders, and grassroots KP-led Civil and Social Organization(CSO) negatively affect access to care for KP. Elevated stigma and discrimination operating at the interpersonal and structural levels have been documented to inversely affect the coverage of existing HIV prevention and treatment services [20]. Studies in Zambia and Malaysia have shown stigma, discrimination, and violence against KP negatively affect the engagement of these groups in HIV prevention and care interventions [21]. Previous studies conducted in Tanzania, Uganda, and Malawi reported negative attitudes toward healthcare providers and denial of services for KP (29-31). Stigma, discrimination, and negative judgment by health providers were cited as reasons for KP avoiding going to health facilities and opting out for self-care for STIs and other conditions [22]. To address this gap, the training of health providers on how to provide KP-friendly services has been identified as a critical need [10]. However, our study found that only 50% of health providers in Tanzania were trained in providing KP-friendly services, with similar training rates (47.2%) observed in Cameroon which also reported limited engagement of KP [23].

Our study reported limited engagement of the community elders, religious leaders, and grassroots KP-led CSOs increases the misunderstanding of KP as a group and their needs and impedes the development of community-led interventions to address stigma and violence [20]. Additionally, extending health services to KP through safe community centers, outreach, and use of KP peers and engaging KP CSO has been shown to improve engagement and offer KP-friendly services [18,21,24-26].

Tanzania has made strides in improving the legal and policy environment for addressing stigma, discrimination, and violence against KP to reduce new HIV infections as part of the national HIV response plan or denial of services [27,28]. Despite progress, the Tanzania penal code still criminalizes sex work, same-sex relationships, and possession of drugs for personal use. Due to fear of persecution, KP hides its risky behaviors, missing appropriate interventions and limiting health providers’ engagement for fear of being interpreted as aiding criminals. Criminalization laws have been shown to alienate and limit the engagement of KP in HIV health care services in Cameroon, Uganda, and Malawi and negatively affect the development of interventions to address stigma [23]. This study also reported arrests among KP, including peers that provide venue-based interventions such as HIV testing and condom provisions. An online global survey conducted in 2012 involving 4020 participants reported about 8% of MSM were ever arrested or convicted for being MSM. Those arrested had significantly lower access to STI treatment, access to condoms, and medical care when compared to other MSM. Rates of reported arrests were highest in sub-Saharan Africa [29]. In different settings, women in possession of condoms were used as evidence for sex work hence arrests [21]. Among PWUD, criminalization, limited harm reduction services, inadequate substance use treatment programs, and HIV services targeting PWID have resulted in higher HIV prevalence [30]. Structural interventions such as engaging law enforcement and referring users to health and social services instead of prison have improved health outcomes among KP [31]. Also, training on human rights and harm reduction for police officers has been shown to improve engagement with KP [31].

**Limitation:** the authors acknowledge several limitations in the study. Non-random sampling may have resulted in the under-representation of some KP groups. Due to stigma and discrimination, some KP might be unwilling to attend the engagement meetings, so opinions of hidden KP were not captured in these meetings. For example, no MSM attended the engagement meeting in the lake zone. The lack of disaggregated data on access to health services by the KP group hindered the authors’ granular analysis from identifying which KP group had comparably poor access to HIV interventions.

## Conclusion

This study clearly showed low engagement of KP in health services and various barriers at different levels affecting engagement and we propose the following interventions; engagement and training of community and religious leaders as the first responders to interpersonal and structural stigma, discrimination, and violence. This can be achieved through community education programs and referrals to the police gender desk and health services. Training packages that include an overview of the HIV epidemic and KP interactions, harm reduction, a human rights-based approach to KP, addressing stigma and discrimination, and referral across existing services should be developed targeting the community, religious, and police force leaders. The aim is to improve community and religious leader engagement and reduce judgmental attitudes and harassment from law enforcement. Additional health providers will need to be trained to offer KP-friendly services using the available national curriculum to improve access to health facilities. These services should be available and manned by skilled staff beyond regular working hours. Also, peers of all typologies (types/groups, e.g. MSM, FSW, PWID) should be available in facilities as KP prefer to be matched to peer navigators.

The Ministry of Health, with technical support from national partners, should develop a comprehensive package for service provision for KP at the community level beyond HIV testing and linkage. The package should include HIV testing services, ART initiation, TB and STI screening, and viral load testing. These services should be available at the selected community points such as safe houses/centers and staffed by peers and trained providers offering friendly and stigma-free services. During outreach activities in the hotspots, community leaders and the police force should be informed of security provisions and support; this will minimize unwarranted arrests and harassment. Efforts to address and prevent stigma should continue through advocacy and sensitization to health providers, law enforcement, and influential leaders. Also, implementing and enforcing anti-discrimination and protective laws is paramount in reducing stigma, discrimination, and violence against people living with HIV(PLHIV) and key populations. TACAIDS, in collaboration with CSO and the Tanzania parliament through its committees (e.g., Parliamentary Committee for HIV/AIDS, TB, and drugs), should review and reform laws that criminalize sex work, possession of drugs for personal use, and same-sex relations.

### What this study adds

- Key populations are at increased risk of HIV due to their risky behaviors and various structural barriers;
- Various factors at different levels are responsible for lower rates of health services utilization among KP;
- Creating social and structural support is known to improve engagement of KP in health services.

### What is known about this topic

- This study shows KP in Tanzania have limited engagement in utilizing health services;
- Key populations friendly services are key to improving access to health services among KP;
- Engaging influential leaders, community and police through advocacy meetings and training is crucial in addressing stigma, discrimination, and violence against KP.
